# Insecure attachment and impaired reflective functioning mediate the association between childhood trauma and grazing behavior

**DOI:** 10.3389/fpsyg.2025.1703521

**Published:** 2025-12-10

**Authors:** Alessandro Alberto Rossi, Andrea Tagliagambe, Stefania Mannarini, Laura Dalla Ragione

**Affiliations:** 1 Department of Philosophy, Sociology, Education, and Applied Psychology, Section of Applied Psychology, University of Padua, Padua, Italy; 2 Center for Intervention and Research on Family Studies—CIRF—Department of Philosophy, Sociology, Education, and Applied Psychology, Section of Applied Psychology, University of Padua, Padua, Italy; 3 Residence Cabrini DCA, Pontremoli, Italy; 4 Food Science and Human Nutrition Unit, University Campus Biomedico of Rome, Rome, Italy

**Keywords:** grazing, addiction-like eating behaviors, childhood traumatic experiences, attachment anxiety, mentalization, reflective functioning, food addiction, eating addiction

## Abstract

**Introduction:**

Grazing represents a clinically significant eating behavior characterized by repetitive consumption of small amounts of food, often associated with poor outcomes in bariatric surgery populations. Despite growing evidence linking childhood traumatic experiences to disordered eating, the specific psychological mechanisms underlying grazing behaviors remain poorly understood. Developmental theories suggest early relational trauma may disrupt attachment patterns and impair emotional self-regulation through mentalization deficits, which could lead to disordered eating behaviors. However, this pathway does not appear to have been tested. This study examined whether attachment insecurity and impaired reflective functioning mediate the relationship between childhood trauma and grazing behaviors.

**Method:**

A cross-sectional study was conducted with 681 Italian adults. Participants completed self-report measures. Structural equation modeling with latent variables and bootstrap resampling (5,000 iterations) was used.

**Results:**

The model demonstrated satisfactory fit and supported the hypothesized pathways. Childhood traumatic experiences were associated with both attachment anxiety and attachment avoidance. However, only attachment anxiety significantly mediated the relationship between childhood trauma and impaired reflective functioning. Impaired reflective functioning emerged as the strongest predictor of grazing behaviors. The complete mediation pathway through attachment anxiety and impaired reflective functioning was statistically significant, with the model explaining 36.4% of variance in grazing behaviors.

**Discussion:**

These findings provide empirical support for a trauma-informed developmental model of grazing behaviors. The results highlight attachment anxiety and impaired reflective functioning as crucial mediators linking early relational trauma to problematic eating patterns. Clinical implications suggest mentalization-based interventions targeting attachment insecurity may be particularly beneficial for individuals with trauma histories presenting with grazing behavior.

## Introduction

Adverse and traumatic relational experiences during childhood (CTEs) are powerful predictors of later mental health problems, including eating disorders and disordered eating behaviors (Linden and LeMoult, 2022; Teicher et al., 2022; Garon-Bissonnette et al., 2023; Pesavento and Rossi, 2025). These experiences—such as emotional maltreatment, neglect, and physical abuse—affect approximately one-third of populations worldwide (Stoltenborgh et al., 2015; Garon-Bissonnette et al., 2022, 2023), representing a significant public health concern. The impact of CTEs can accumulate over time, with effects persisting from early development into adulthood (Schimmenti, 2018; Musetti et al., 2023). Notably, psychological forms of childhood maltreatment, including emotional abuse and neglect (Baker and Maiorino, 2010; Pesavento and Rossi, 2025), have proven particularly damaging, with consequences comparable to those following physical or sexual abuse (Vachon et al., 2015).

Recent literature emphasizes that both physical and psychological CTEs play a crucial role in the emergence of eating disorders and disordered eating behaviors, including overeating and food addiction (Brewerton, 2007, 2017; Guney et al., 2025; Rossi et al., 2025a; Rossi and Mannarini, 2025; Wiss and LaFata, 2025). Large-scale studies further indicate that individuals with histories of CTEs face substantially elevated risks for developing various forms of disordered eating, with emotional neglect showing particularly strong associations with problematic eating patterns (Emery et al., 2021; Musetti et al., 2023). Consistently, CTEs has been reported at higher rates both in clinical populations with eating disorders and in individuals from the general population exhibiting disordered eating behaviors, compared to healthy samples (Hicks White et al., 2018; Groth et al., 2020; Quattropani et al., 2022). Moreover, greater exposure to such early-life trauma is associated with increased severity of eating-related symptoms (Backholm et al., 2013).

However, evidence suggests that the link between CTEs and subsequent mental health problems, particularly eating-related disorders, often operates through complex indirect mechanisms rather than direct causal pathways (Schimmenti, 2018; Musetti et al., 2022, 2023; Rossi et al., 2025a). In particular, insecure attachment and impaired self-regulation may serve as bridges between CTEs and disordered eating behaviors (Rossi et al., 2025a; Santoro et al., 2025a, 2025b).

Substantial research evidence indicates that exposure to CTEs significantly increases the likelihood of developing insecure attachment patterns, which subsequently elevate risk for various psychological disorders (Schimmenti and Caretti, 2016; Schimmenti, 2018; Midolo et al., 2020). From a developmental perspective, early caregiver–child interactions establish foundational templates for future relational experiences (Cassidy and Shaver, 2018; Thompson, 2024; Nielsen et al., 2025). The attachment system functions as a primary motivational framework through which individuals seek protection and comfort in relationships (Bowlby, 1988). These early interactions form enduring cognitive–emotional templates that, even in adulthood, guide the ability to self-regulate emotions and behaviors within both intrapersonal and interpersonal contexts (Bowlby, 1973). When primary relationships lack security and safety, individuals may experience profound difficulties in establishing meaningful connections with self and others throughout their life span (Davila et al., 2005; Musetti et al., 2022, 2023).

According to the literature, insecure attachment patterns can be conceptualized through two fundamental dimensions that capture individual differences in attachment functioning: attachment avoidance and attachment anxiety (Bartholomew and Horowitz, 1991; Bartholomew and Shaver, 1998; Mikulincer and Shaver, 2003). Individuals with attachment avoidance tend to hold positive self-regard while viewing others negatively, resulting in defensive strategies that involve emotional distancing and discomfort with intimacy (Mikulincer et al., 2003). In contrast, individuals with attachment anxiety typically harbor negative self-concepts while maintaining positive views of relationship partners, leading to excessive reliance on others, coupled with intense fears of rejection or abandonment. This dynamic undermines the development of a stable internal psychological structure, making individuals more prone to dysregulated thoughts, emotions, behaviors, and interpersonal functioning capacities such as reflective functioning (Rossi et al., 2025a; Rossi and Mannarini, 2025; Santoro et al., 2025b).

These two attachment dimensions operate through fundamentally distinct regulatory strategies with differential impacts on reflective functioning and emotion regulation. Individuals with attachment anxiety employ hyperactivating strategies characterized by heightened emotional reactivity, excessive preoccupation with relational cues, and intense fear of rejection—creating a state of chronic emotional flooding that directly overwhelms mentalizing capacity, particularly under stress (Mikulincer et al., 2003; Long et al., 2020). This hypervigilance to relational threats makes it difficult to maintain balanced, reflective understanding of one’s own and others’ mental states (Musetti et al., 2022, 2023). Conversely, individuals with attachment avoidance utilize deactivating strategies involving emotional suppression, defensive self-reliance, and interpersonal distancing (Mikulincer et al., 2003). While these strategies may preserve some capacity for cognitive mentalizing through emotional detachment, they often result in pseudomentalizing—a superficial understanding of mental states that lacks genuine affective grounding and authentic emotional engagement (Sheinbaum et al., 2015; Luyten et al., 2020). Consequently, attachment anxiety appears more directly disruptive to reflective functioning compared to attachment avoidance, a differential pattern consistently observed in eating disorder populations (Tasca, 2019; Jewell et al., 2023).

Empirical investigations have suggested that attachment anxiety often accounts for the relationship between traumatic experiences and the severity of eating pathology (Meltzer-Brody et al., 2011; Tasca et al., 2013; Tasca, 2019; Solmi et al., 2020; Musetti et al., 2023). Consequently, attachment anxiety has been consistently identified as a vulnerability factor for both impairments in self-regulation and interpersonal functioning, as well as for eating-related difficulties (Jewell et al., 2016, 2023; Midolo et al., 2020; Musetti et al., 2023; Santoro et al., 2025b).

From a developmental perspective, insecure attachment—particularly attachment anxiety—is strongly associated with impairments in reflective functioning. Rooted in early attachment relationships (Fonagy et al., 1991, 2002) and broader environmental influences (Allen et al., 2008; Luyten et al., 2020), reflective functioning represents the operationalization of mentalization (Fonagy et al., 1998; Katznelson, 2014; Luyten et al., 2020) and refers to the ability to understand and interpret human behavior through underlying psychological states such as emotions, thoughts, and motivations (Fonagy et al., 2002). This psychological capacity involves the integration of both automatic and deliberate mental processes, encompassing cognitive and emotional abilities directed toward understanding both oneself and others (Katznelson, 2014; Bateman and Fonagy, 2019). More specifically, the broader construct of mentalization encompasses multiple dimensions—including automatic versus controlled processing, cognitive versus affective focus, self versus other orientation, and internal versus external focus (Luyten et al., 2020)—while reflective functioning specifically captures the capacity to explicitly reflect on mental states. Impairments in reflective functioning can manifest as hypomentalization (excessive uncertainty about mental states) or hypermentalization (excessive certainty) (Fonagy et al., 2016). Hypomentalization reflects a reduced capacity to accurately recognize mental state complexity, characterized by excessive uncertainty about psychological processes (Fonagy et al., 2016). In contrast, hypermentalization involves excessive certainty and overinterpretation of mental states, leading to rigid and often inaccurate attributions (Fonagy et al., 2016). Among these impairments, hypomentalization has demonstrated particularly robust associations with attachment insecurity and eating pathology (Musetti et al., 2023; Rossi et al., 2025a, 2025b; Santoro et al., 2025a), making it a critical focus for understanding maladaptive eating behaviors.

When functioning optimally, reflective functioning enables individuals to make sense of behavioral patterns and serves as a crucial component of emotional regulation processes (Fonagy et al., 1998; Asen and Fonagy, 2017; Garon-Bissonnette et al., 2023), thereby supporting overall psychological adaptation (Fonagy et al., 2002; Macintosh, 2013; Garon-Bissonnette et al., 2023). Conversely, when reflective functioning is impaired through hypomentalization, individuals experience persistent difficulties in understanding their own and others’ mental states, leading to defensive withdrawal from psychological reflection as self-protection from overwhelming emotional experiences (Cicchetti and Toth, 2005; Allen et al., 2008; Lorenzini et al., 2019). These impairments result in compromised emotional regulation capacities and increased vulnerability to maladaptive coping strategies.

Research has demonstrated that uncertainty about mental states partially mediates the relationship between childhood emotional maltreatment, attachment patterns, and psychological symptoms (Santoro et al., 2021), as well as problematic eating behaviors (Gagliardini et al., 2020; Musetti et al., 2023; Rossi and Mannarini, 2025; Rossi et al., 2025a). Indeed, several studies have identified elevated impaired reflective functioning among individuals with eating disorders compared to healthy controls (Cucchi et al., 2018; Sarig-Shmueli et al., 2023; Kjaersdam Telléus et al., 2024; Santoro et al., 2025a).

When individuals cannot adequately understand or regulate their internal emotional states due to impaired reflective functioning, they may turn to food-related behaviors as alternative strategies for managing psychological distress (Musetti et al., 2023; Gagliardini et al., 2024; Rossi and Mannarini, 2025; Rossi et al., 2025a; Santoro et al., 2025a). The use of food becomes a means of regulating overwhelming mental states when more sophisticated psychological mechanisms are compromised, potentially leading to problematic eating patterns such as emotional eating, binge eating (Rossi and Mannarini, 2025), or grazing behaviors.

Grazing has been increasingly recognized as a maladaptive eating behavior often used to cope with or regulate intense and predominantly negative emotions (Conceição et al., 2014a). Strongly related to food addiction and addictive-like eating behaviors (Rossi et al., 2024), individuals engaging in grazing often report an irresistible urge to eat (Ribeiro et al., 2023; Rossi et al., 2023a), leading to compulsive overeating and loss of control over highly processed, hyper-palatable foods (Gearhardt and Schulte, 2021; Parnarouskis and Gearhardt, 2022; Gearhardt and DiFeliceantonio, 2023; LaFata et al., 2025), without necessarily assuming the shape of binge eating. Indeed, grazing is characterized by the unplanned, repetitive intake of small to moderate amounts of food throughout the day, typically occurring outside of structured meals (Conceição et al., 2014a, 2017; Heriseanu et al., 2019; Conceição et al., 2023). Although grazing is not currently classified as a distinct eating disorder in the DSM-5, it represents a clinically significant dimensional eating behavior associated with loss of control, psychological distress, and poor treatment outcomes, particularly in bariatric surgery populations (Colles et al., 2008; Conceição et al., 2014a, 2017).

In this regard, Conceição et al., 2014a proposed a distinction between two forms of grazing. Non-compulsive grazing, also referred to as repetitive eating, reflects a distracted pattern of eating that generally occurs without marked cognitive or emotional involvement and is associated with minimal loss of control. In contrast, compulsive grazing involves a subjective sense of being unable to resist food, with repeated urges to eat despite attempts to refrain, reflecting greater loss of control and a stronger link to binge eating episodes (Conceição et al., 2014b, 2014a, 2017). Compulsive grazing has been associated with heightened psychological distress, including negative affect, anxiety, and depressive symptoms, as well as poorer overall mental health (Poole et al., 2005; Colles et al., 2008; Conceição et al., 2017). It has also been linked to higher body mass index, greater hunger, reduced dietary inhibition (Colles et al., 2008), and, importantly, poorer postoperative outcomes in bariatric surgery, where it emerges as a leading predictor of insufficient weight loss or weight regain (Colles et al., 2008; Conceição et al., 2014b, 2015; Mechanick et al., 2020). In many cases, patients with pre-surgical binge eating tendencies appear to shift toward grazing after surgery, particularly when physical restrictions limit the capacity for large-volume eating (Colles et al., 2008; Conceição et al., 2017).

Consequently, these intertwined mechanisms highlight the importance of grazing not only as a behavioral marker of emotional dysregulation but also as a clinically relevant target in the prevention and treatment of obesity and eating disorders, especially in the context of bariatric surgery and food addiction-related pathology.

However, despite growing evidence linking CTEs, attachment-related processes, and maladaptive eating behaviors, the specific role of reflective functioning in the emergence of compulsive grazing remains underexplored. The present study therefore aims to investigate whether impairments in reflective functioning mediate the relationship between early adverse relational experiences, attachment anxiety, and compulsive grazing behaviors, contributing to a better understanding of the psychological mechanisms underlying this addictive-like eating patterns.

Building on the aforementioned literature, the present research set out to examine a multiple-mediation framework. For a comprehensive overview of the key constructs and supporting empirical evidence discussed in this introduction, see Supplementary Table S1. Specifically, we proposed that childhood traumatic experiences (CTEs; X) would be linked to two dimensions of insecure attachment—attachment anxiety (M1a) and attachment avoidance (M1b)—as well as to impaired reflective functioning (M2). impaired reflective functioning (M2) was then expected to relate to grazing (Y).

Accordingly, the following hypotheses were formulated:

- H1: All key variables – CTEs, attachment anxiety, attachment avoidance, impaired reflective functioning, and grazing – would show positive intercorrelations.
- H2: The relationship between CTEs and impaired reflective functioning would be explained, at least in part, by attachment anxiety
- H3: The link between CTEs and grazing would be mediated by attachment anxiety, attachment avoidance, and impaired reflective functioning.

Thus, it was expected that early traumatic relational experiences would be associated with grazing behaviors through a cascade of interconnected psychological processes, including insecure attachment and impaired reflective functioning.

## Methods and materials

### Sample size determination

In line with previous studies, the number of participants was determined in advance according to the “*n:q criterion*,” which considers the ratio between the sample size (*n*) and the number of model parameters (*q*) (Brown, 2015; Kline, 2023). To achieve adequate statistical power, a ratio of 10 participants per parameter was set. Given a total of 49 parameters (see statistical analysis section), this led to a minimum required sample of 490 individuals.

### Procedure

According to previous studies, participants were drawn from the general population using a convenience sampling method, specifically snowball sampling (Fricker, 2012), via social media platforms such as Facebook and X (formerly Twitter). Eligibility criteria included: (A) being 18 years of age or older; (B) native proficiency in Italian; (C) complete responses to the assessment battery (i.e., no missing data); (D) provision of informed consent.

In addition, consistent with current best practices, data quality checks were performed before conducting statistical analyses to ensure the validity of the online responses (Pozzar et al., 2020). Participants were informed in advance that no compensation would be provided. All participants took part voluntarily and provided informed consent. The study received approval from the Ethics Committee of the University of Padua (protocol no. 547-a) and adhered to the institution’s ethical guidelines.

### Participants

The sample comprised 681 participants. The sample included 128 males (18.8%) and 553 females (81.2%), aged between 18 and 70 years (*mean* = 41.59, *SD* = 14.19), with a BMI ranging from 16.53 to 55.10 kg/m^2^ (*mean* = 24.23 kg/m^2^, *SD* = 4.91). Specifically, 583 participants (85.6%) reported no prior history of eating disorders, while 98 participants (14.4%) reported a positive history, distributed as follows: 25 (3.7%) self-reported a diagnosis of Anorexia Nervosa (AN), 36 (5.3%) Bulimia Nervosa (BN), 25 (3.7%) Binge Eating Disorder (BED), and 10 (1.4%) Otherwise Specified Feeding and Eating Disorder (OSFED). More details are provided in Table 1.

**Table 1:** Sample descriptive statistics.

Descriptives	(*N* = 681)	
Sex (*n*, %)		
Male	128	18.8%
Female	553	81.2%
Education (*n,* %)		
Middle school	57	8.4%
High school	308	45.2%
Bachelor	260	38.2%
Master degree/PhD	56	8.2%
Work status (*n,* %)		
Student	108	15.9%
Full-time worker	299	43.9%
Part-time worker	94	13.8%
Entrepreneur	76	11.2%
Housewife	25	3.7%
Unemployed	27	4.0%
Retired	52	7.6%
Civil status (*n*, %)		
Single	172	25.3%
In a relationships	185	27.2%
Married	250	36.7%
Separated/divorced	62	9.1%
Widowed	12	1.8%
BMI class		
Underweight (16–18.49)	39	5.7%
Normal weight (18.5–24.99)	402	59.0%
Overweight (25–29.99)	159	23.3%
Class I obesity (30–34.99)	61	9.0%
Class II obesity (35–39.99)	12	1.8%
Class III obesity (> 40)	8	1.2%
Eating disorder (*n*. %)		
No ED	583	85.6%
Anorexia Nervosa (AN)	25	3.7%
Bulimia Nervosa (BN)	36	5.3%
Binge Eating Disorder (BED)	25	3.7%
Otherwise specified feeding and eating disorder (OSFED)	12	1.7%

### Measures

#### Childhood trauma questionnaire–short form (CTQ-SF)

The CTQ-SF (Bernstein et al., 2003) is a self-report questionnaire assessing adverse and traumatic childhood experiences. It measures five different kind of relational CTEs: emotional abuse, physical abuse, sexual abuse, emotional neglect, and physical neglect. It consists of 28 items rated on a 5-point Likert scale. A total score, obtained by summing the subscale scores, reflects overall childhood trauma, with higher scores indicating more severe experiences. For this study, the validated Italian version of the CTQ-SF (Sacchi et al., 2018) was used, showing excellent internal consistency (McDonald’s ω = 0.945).

#### Relationship questionnaire (RQ)

The RQ (Bartholomew and Horowitz, 1991) is a self-report tool for assessing adult attachment derived from early relationships with the primary caregiver (Mikulincer and Shaver, 2003; Mikulincer et al., 2003). Participants rate how well four brief descriptions match their typical relationship style on a 7-point Likert scale. Each description represents one of four prototypical attachment styles: secure, dismissing, preoccupied, and fearful. Following standard scoring (Bartholomew and Horowitz, 1991), for the present study, two core insecure attachment dimensions were computed: anxiety [(fearful + preoccupied)−(secure + dismissing)] and avoidance [(fearful + dismissing)−(secure + preoccupied)]. Since each dimension is calculated by combining single-item prototype ratings using a difference score formula, conventional internal consistency reliability estimates (e.g., McDonald’s omega) cannot be meaningfully applied to this scoring approach. Higher scores reflect greater attachment anxiety or avoidance. This study used the validated Italian version of the RQ (Carli, 1995).

#### Reflective functioning questionnaire (RFQ)

The RFQ (Fonagy et al., 2016) is a short self-report tool assessing reflective functioning (Fonagy et al., 2002). It consists of eight items rated on a 7-point Likert scale. The measure captures two opposing aspects: certainty (hypermentalizing) and uncertainty (hypomentalizing) about mental states. In this study, only the uncertainty subscale was used, as it represents—to date—the closest assessment of hypomentalization, with higher scores reflecting greater impairment in reflective functioning. The validated Italian version (Morandotti et al., 2018) was administered and showed good internal consistency (McDonald’s ω = 0.894).

#### The repetitive eating questionnaire (rep(eat)-Q)

The Rep(Eat)-Q (Conceição et al., 2017, 2023) is a brief self-report questionnaire assessing the frequency of grazing-related attitudes and behaviors over the past 28 days. The questionnaire evaluates two facets of grazing: repetitive eating and compulsive grazing. Repetitive eating, reflecting distracted, disorganized, and unaware eating often linked to binge eating. Compulsive grazing, capturing small or moderate food intake driven by irresistible urges and associated distress. The Rep(Eat)-Q contains 12 items rated on a 7-point Likert scale, with higher scores indicating more frequent grazing. Also, a general overall score could be computed. In line with literature, considering, the high correlation between the two subscale, only the total score was used in the present study. The Italian version (Rossi et al., 2024) of the questionnaire was administered and showed good internal consistency (McDonald’s ω = 0.963).

### Statistical analysis

All statistical analyses were conducted in R (v 4.3.2). The following packages were used: psych (v. 1.8.12; Revelle, 2018), lavaan (v. 0.6–12; Rosseel, 2012), semTools (v. 0.5–2; Jorgensen et al., 2022), and Tidyverse (v. 2.0.0 Wickham et al., 2019). Graphical representations were performed with ggplot2 (v. 351; Wickham, 2016). As per the predefined inclusion criteria, individuals with missing data were excluded form the analysis—thus, the dataset contained no missing values. Following previous recommendations (McDonald and Ho, 2002; Rucker et al., 2011), several preparatory steps were carried out prior to estimating the structural equation model (SEM).

As an initial step, Pearson’s correlation coefficients (*r*) were computed to assess the strength of associations among variables and detect potential concerns. Following guidelines, correlations were classified as trivial (*r* < 0.10), small (0.10–0.30), moderate (0.30–0.50), or large (>0.50) (Cohen, 1988). Correlations above |0.80| were considered suggestive of problematic overlap (Tabachnick and Fidell, 2019; Rönkkö and Cho, 2022; Panzeri et al., 2024b).

Next, the measurement model (i.e., the factorial structure) of each questionnaire was examined using confirmatory factor analysis (CFA) to test whether the data aligned with the theoretical models from prior validation studies. According to literature, the RQ was excluded, as its original validation does not support a meaningful factorial evaluation. CFAs were conducted using the diagonally weighted least squares (DWLS) estimator (Brown, 2015; Kline, 2023). Model fit was judged according to established criteria (Hu and Bentler, 1999): (A) a non-significant χ^2^ (*p* > 0.05) was preferred; (B) RMSEA < 0.08 indicated acceptable fit; (C) CFI > 0.90 denoted satisfactory fit; and (D) SRMR < 0.08 reflected good fit.

Moreover, Harman’s single-factor test (Harman, 1976; Podsakoff et al., 2003) was used to evaluate potential common method bias, testing whether a single latent factor accounted for most of the covariance among variables. First, a model with correlated latent factors was specified based on the theoretical structure of the scales. Then, a constrained model was estimated with all items loading onto a single factor. Model comparisons relied on standard fit thresholds (van de Schoot et al., 2012): a nonsignificant χ^2^ change (Δχ^2^, *p* > 0.05), along with ΔCFI ≤ 0.01 and ΔRMSEA ≤ 0.015, indicated no meaningful deterioration. A superior fit of the correlated-factor model relative to the single-factor model would suggest that common method bias was unlikely.

Furthermore, latent variables were represented via item parcels as indicators (Little et al., 2013). According to previous research (Coffman and MacCallum, 2005), a mixed item-parceling strategy was used. Specifically, for the ‘RFQ uncertainty’ and the ‘Rea(Eat)-Q’, parcels were constructed via the ‘item-to-construct balance’ method (Little et al., 2002, 2013)—ensuring a minimum of three item-parcels to guarantee model just-identification (Mackinnon, 2012; Little et al., 2013; Kline, 2023). For the CTQ-SF, item-parcels were created via the ‘domain-representative strategy’ (Little et al., 2002, 2013). No item parcels were created for the two dimensions of RQ, as they consists of a single item/dimension each. Consequently, to retain its theoretical meaning, a single-item latent variable was modeled using the reliability correction method (Bollen, 1989; Coffman and MacCallum, 2005; Williams and O’Boyle, 2008). Following Jorgensen (2025) and established guidelines, each single-item indicator’s residual variance was set to the item’s total variance multiplied by 1 − *α*, where α represents internal consistency (Jorgensen, 2025). The reliability coefficient (α) was fixed *a priori* at 0.75^1^, consistent with conventional recommendations for single-item measures (Nunnally, 1978; Jöreskog and Sörbom, 1996; Hayduk and Littvay, 2012).

**Figure 1:**
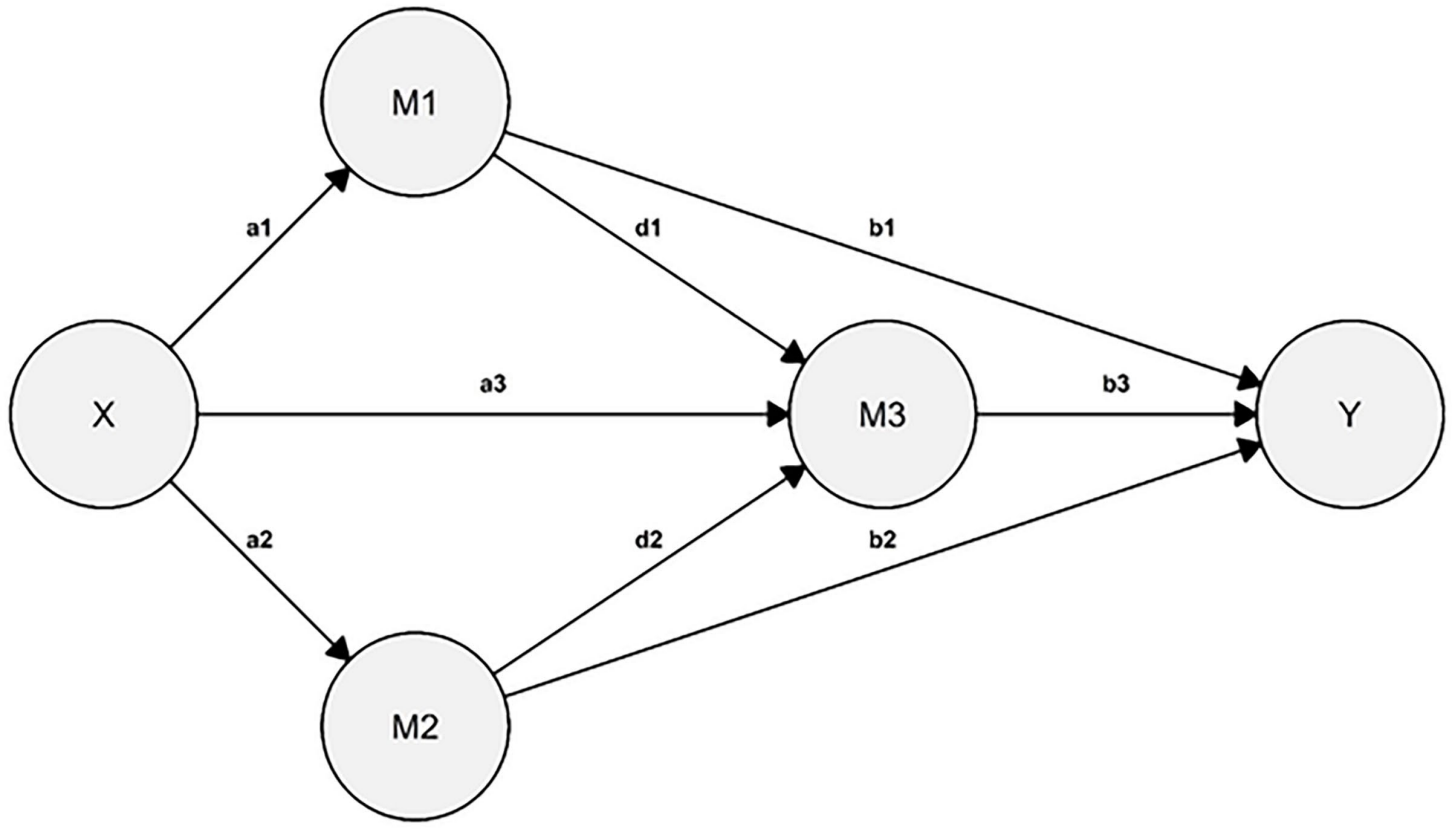
SEM conceptual representation. Sex and BMI were included as covariates. For the sake of clarity in the presentation of this graph, covariates, item parcels, and indirect effects (e.g., X → Y) were not included; only direct effects were depicted. The circles represent latent variables.

Lastly, a SEM with latent variables was estimated, including one predictor, three mediators, and one outcome (Iacobucci, 2009; Mackinnon, 2012). Childhood traumatic experiences (X) was specified as predicting grazing (Y), with attachment anxiety (M1), attachment avoidance (M2), and impairment in reflective functioning (M3) as mediators (see Figure 1). Sex and BMI were included as covariates. The model was estimated using maximum likelihood (ML), and because some indicators deviated from normality, a nonparametric bootstrap with 5,000 resamples and Bollen–Stine correction was applied (Bollen and Stine, 1993; MacKinnon et al., 2013; Kline, 2023). Fit was evaluated using aforementioned goodness of fit indices (χ^2^, RMSEA, CFI, and SRMR) and their conventional thresholds (Hoyle, 2023; Kline, 2023). All reported regression coefficients (*β*) are unstandardized.

## Results

### Preliminary analysis

Correlation analyses (H1) revealed associations ranging from small to large among the psychological variables included in the mediation model (Table 2). Furthermore, examining continuous covariates include in the model, the correlation matrix indicated minimal effects of BMI.

**Table 2:** Correlations among variables.

		Descriptives				Correlations					
		M	SD	Sk	K	1	2	3	4	5	6
1	Childhood Trauma	37.57	12.79	1.72	3.32	–					
2	Attachment anxiety	−0.84	4.42	0.33	−0.17	0.174^***^	–				
3	Attachment avoidance	0.68	3.53	0.17	−0.28	0.146^***^	0.152^***^	–			
4	Impaired reflective functioning	2.78	4.17	1.81	2.70	0.094^*^	0.533^***^	0.128^**^	–		
5	Grazing	1.59	1.44	0.98	0.00	0.140^***^	0.376^***^	0.100^**^	0.504^***^	–	
6	BMI	24.24	4.91	1.41	3.26	0.060	−0.101^**^	−0.017	−0.064	0.155^***^	–

***p < 0.001; **p < 0.010. *p < 0.050. M, mean; SD, standard deviation; Sk, skewness; K, kurtosis. Childhood Trauma, CTQ-SF Total score; Attachment anxiety, attachment anxiety dimension of the RQ; Attachment avoidance, attachment avoidance dimension of the RQ; Impaired reflective functioning, uncertainty scale of the RFQ; Grazing, Rep(Eat)-Q total score; BMI, body mass index.

### Measurement models—factorial structure analysis

The measurement models of all questionnaires used in the SEM showed satisfactory fit indices. The CTQ-SF demonstrated satisfactory fit: χ^2^(270) = 713.866, *p* < 0.001; RMSEA = 0.049, 90% CI [0.045, 0.053], *p*(RMSEA < 0.05) = 0.615; CFI = 0.995; SRMR = 0.076. Similarly, the RFQ uncertainty scale showed acceptable fit: χ^2^(9) = 40.864, *p* < 0.001; RMSEA = 0.072, 90% CI [0.051, 0.095], *p*(RMSEA < 0.05) = 0.046; CFI = 0.996; SRMR = 0.055. The Rep(Eat)-Q also displayed adequate fit: χ^2^ (53) = 211.099, *p* < 0.001; RMSEA = 0.066, 90% CI [0.057, 0.066], *p*(RMSEA < 0.05) = 0.002; CFI = 0.999; SRMR = 0.031. As previously noted, assessing the factorial structure of the RQ is not meaningful.

### Harman’s single-factor test

The single-factor test indicated no evidence of common method bias. The model with correlated factors showed good fit: χ^2^(832) = 1567.325, *p* < 0.001; RMSEA = 0.036, 90% CI [0.033, 0.039], *p*(RMSEA < 0.05) = 1; CFI = 0.997; SRMR = 0.066. By contrast, the single-factor model exhibited poor fit: χ^2^(860) = 50910.614, *p* < 0.001; RMSEA = 0.293, 90% CI [0.290, 0.295], *p*(RMSEA < 0.05) < 0.001; CFI = 0.784; SRMR = 0.395. Comparison of the two models confirmed the absence of common method bias: Δχ^2^(28) = 49,343, p < 0.001; |ΔRMSEA| = 0.256; |ΔCFI| = 0.213.

### Structural equation model with latent variables and item parcels

**Figure 2:**
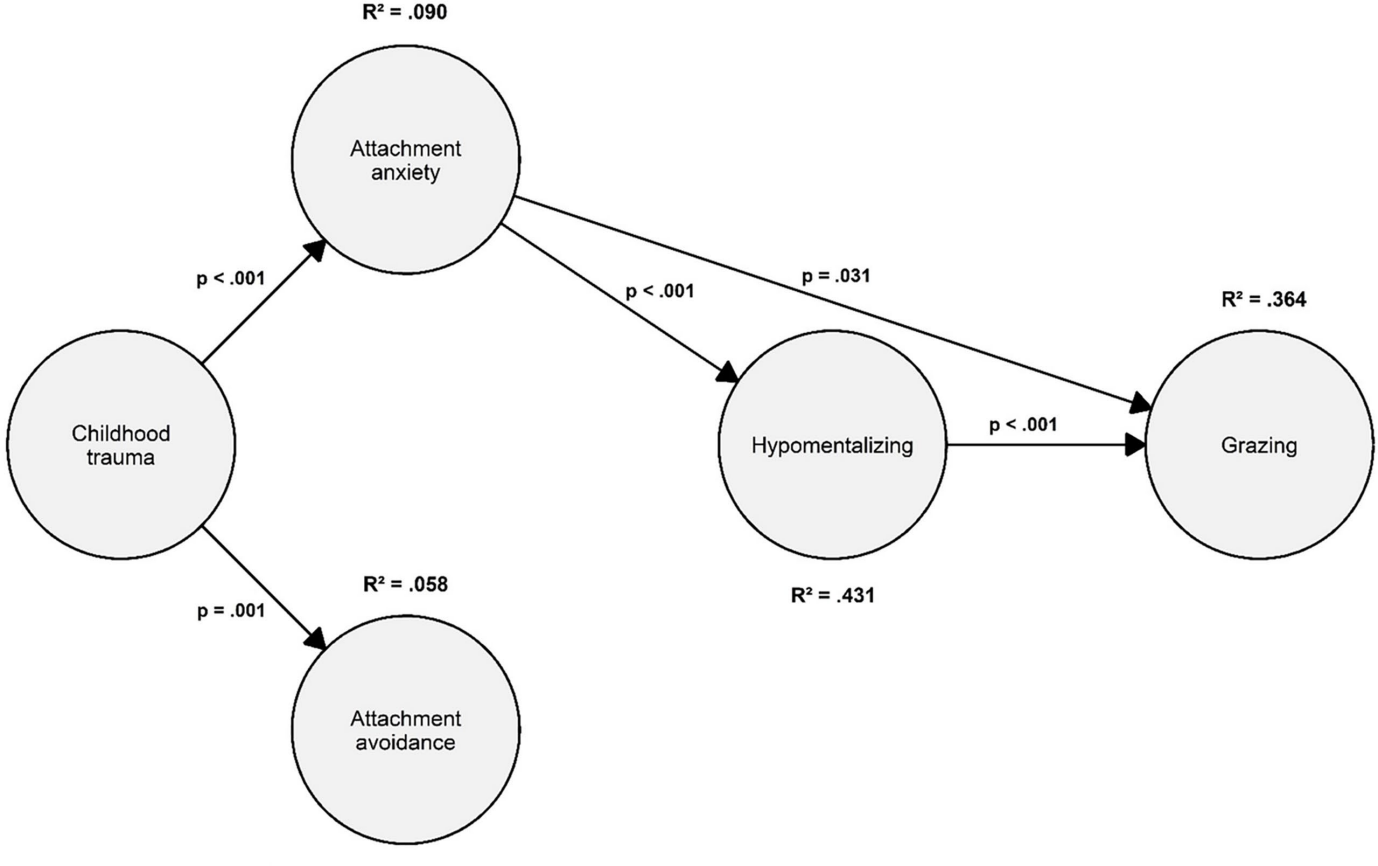
Model results. Sex and BMI were included as covariates. For the sake of clarity in the presentation of this graph, covariates, item parcels, and indirect effects (e.g., X → Y) were not included; only direct effects were depicted. The circles represent latent variables. *p* = *p* value. R^2^ = explained variance.

The hypothesized SEM (Figures 1, 2) demonstrated satisfactory fit: χ^2^(89) = 304.380, *p* < 0.001; RMSEA = 0.060, 90% CI [0.052, 0.067], *p*(RMSEA < 0.05) = 0.015; CFI = 0.968; SRMR = 0.035. All items were significantly associated with their respective latent variables (Table 3). To maintain clarity, only sequential paths (e.g., X → M1, M1 → M3) are reported here; full results are presented in Tables 4, 5.

**Table 3:** Descriptive statistics and factor loadings (*λ*) of the item parcels.

	Descriptives				Item parcel/latent variable relationship			
	M	SD	Sk	K	λ(se)	*z* value	*p* value	λ*
Childhood trauma (X)								
pCTQ#1	1.35	0.48	2.08	5.87	1.000			0.717
pCTQ#2	1.67	0.86	1.57	2.08	2.058 (0.157)	13.089	< 0.001	0.832
pCTQ#3	2.16	0.96	0.74	−0.27	2.336 (0.183)	12.769	< 0.001	0.843
pCTQ#4	1.18	0.48	3.74	15.46	0.872 (0.092)	9.516	< 0.001	0.626
pCTQ#5	1.16	0.53	4.19	18.88	0.511 (0.109)	4.667	< 0.001	0.335
Attachment anxiety (M1)								
Att. Anx.	−0.84	4.42	0.33	−0.17	1.000			0.866
Attachment avoidance (M2)								
Att. Av.	0.68	3.53	0.17	−0.28	1.000			0.865
Impaired reflective functioning (M3)								
pRFQ#1	0.37	0.67	2.06	3.83	1.000			0.826
pRFQ#2	0.48	0.79	1.63	1.74	1.242 (0.060)	20.709	< 0.001	0.875
pRFQ#3	0.53	0.84	1.58	1.43	1.298 (0.066)	19.811	< 0.001	0.859
Appetite drive (Y)								
pREP#1	1.60	1.49	0.96	−0.02	1.000			0.939
pREP#2	1.42	1.48	1.06	0.15	0.996 (0.019)	52.296	< 0.001	0.945
pREP#3	1.87	1.55	0.72	−0.46	1.038 (0.021)	49.505	< 0.001	0.938
pREP#4	1.49	1.51	1.03	0.11	1.006 (0.020)	50.668	< 0.001	0.937

M, mean; SD, standard deviation; Sk, skewness; K, kurtosis; λ, unstandardized factor loading; se, standard error; λ*, standardized factor loading; p(…), item parcel; childhood trauma, CTQ-SF total score; attachment anxiety, attachment anxiety dimension of the RQ; attachment avoidance, attachment avoidance dimension of the RQ; Impaired reflective functioning, RFQ uncertainty scale; Grazing, Rep(Eat)-Q total score.

**Table 4:** Summary of simple regression parameter estimates (β) with 95% confidence intervals (Figure 2).

	*path*	β*	β (SE)	95%CI [L - U]	*z value*	*p* value	*R* ^2^
Childhood trauma (X) → Attachment Anxiety (M1)	a1	0.229	2.526 (0.559)	[1.486; 3.691]	4.521	< 0.001	0.090
Childhood trauma (X) → Attachment Avoidance (M2)	a2	0.157	1.379 (0.427)	[0.537; 2.255]	3.227	0.001	0.058
Attachment Anxiety (M1) → Imp. Reflect. Funct. (M3)	d1	0.666	0.097 (0.009)	[0.079; 0.115]	10.421	< 0.001	0.431
Attachment Avoidance (M2) → Imp. Reflect. Funct. (M3)	d2	0.043	0.008 (0.008)	[−0.008; 0.025]	0.961	0.336	
Imp. Reflect. Funct. (M3) → Grazing (Y)	b3	0.456	1.147 (0.160)	[0.849; 1.471]	7.188	< 0.001	0.364
Childhood trauma (X) → Grazing (Y)	c1	0.055	0.223 (0.158)	[−0.095; 0.534]	1.417	0.157	
Attachment Anxiety (M1) ↔ Attachment Avoidance (M2)		0.145	1.564 (0.489)	[0.606; 2.522]	3.201	0.001	
Childhood trauma (X) → Imp. Reflect. Funct. (M3)	a3	−0.049	−0.079 (0.070)	[−0.218; 0.059]	−1.123	0.261	
Attachment Anxiety (M1) → Grazing (Y)	b1	0.139	0.051 (0.024)	[0.007; 0.099]	2.161	0.031	
Attachment Avoidance (M2) → Grazing (Y)	b2	−0.013	−0.006 (0.020)	[−0.047; 0.034]	−0.288	0.774	
Sex ↔ BMI		−0.068	−0.131 (0.061)	[−0.252; −0.011]	−2.146	0.032	
Sex → Attachment Anxiety (M1)		0.140	1.364 (0.434)	[0.497; 2.211]	3.143	0.002	
Sex → Attachment Avoidance (M2)		0.181	1.408 (0.315)	[0.768; 2.013]	4.464	< 0.001	
Sex → Imp. Reflect. Funct. (M3)		−0.076	−0.108 (0.054)	[−0.215; −0.005]	−2.005	0.045	
Sex → Grazing (Y)		0.134	0.478 (0.109)	[0.262; 0.688]	4.401	< 0.001	
BMI → Attachment Anxiety (M1)		−0.124	−0.097 (0.030)	[−0.158; −0.038]	−3.196	0.001	
BMI → Attachment Avoidance (M2)		−0.018	−0.011 (0.028)	[−0.064; 0.043]	−0.410	0.682	
BMI → Imp. Reflect. Funct. (M3)		0.011	0.001 (0.004)	[−0.007; 0.010]	0.299	0.765	
BMI → Grazing (Y)		0.209	0.059 (0.010)	[0.039; 0.080]	5.703	< 0.001	

β*, standardized beta; β, unstandardized beta; SE, standard error; 95% CI, 95% confidence intervals for the unstandardized beta; R^2^, explained variance.

**Table 5:** Summary of parameter estimates for the model’s indirect and total effects (Figure 2).

	*path*	β*	β (SE)	95%CI [L - U]	*z value*	*p* value
Effect of X on Y via M1	a1*b1	0.032	0.129 (0.066)	[0.016; 0.274]	1.944	0.052
Effect of X on Y via M2	a2*b2	−0.002	−0.008 (0.030)	[−0.071; 0.051]	−0.271	0.787
Effect of X on Y via M3	a3*b3	−0.022	−0.091 (0.083)	[−0.263; 0.068]	−1.098	0.272
Effect of X on Y via M1 and M3	a1*d1*b3	0.070	0.281 (0.077)	[0.149; 0.451]	3.656	< 0.001
Effect of X on Y via M2 and M3	a2*d2*b3	0.003	0.013 (0.014)	[−0.013; 0.044]	0.878	0.380
Total indirect effect of X on Y		0.080	0.323 (0.117)	[0.107; 0.565]	2.766	0.006
Total effect on Y		0.136	0.546 (0.170)	[0.223; 0.893]	3.216	0.001

β*, standardized beta; β, unstandardized beta; SE, standard error; 95% CI, 95% confidence intervals for the unstandardized beta. X, childhood trauma; M1, attachment anxiety; M2, attachment avoidance; M3, Impaired reflective functioning; Y, grazing.

Childhood trauma (X) was positively associated with attachment anxiety (M1), *path a1*: β = 2.526 (SE = 0.559), 95% CI [1.486, 3.691]; and with attachment avoidance (M2), *path a2*: β = 1.379 (SE = 0.427), 95% CI [0.537, 2.255]. However, X was not linked to impaired reflective functioning (M3), *path a3*: β = −0.079 (SE = 0.070), 95% CI [−0.218, 0.059]—suggesting a mediating effect of attachment.

Attachment anxiety (M1) was positively associated with impaired reflective functioning (M3), *path d1*: β = 0.097 (SE = 0.009), 95% CI [0.079, 0.115], whereas attachment avoidance (M2) was not significantly related to impaired reflective functioning (M3), *path d2*: β = 0.008 (SE = 0.008), 95% CI [−0.008, 0.025]—revealing the mediating effect of attachment anxiety on the relationship between X and M3. Also, M1 and M2 were modestly positively correlated: β = 1.564 (SE = 0.489), 95% CI [0.606, 2.522].

Lastly, impaired reflective functioning (M3) was positively related to grazing (Y), *path b3*: β = 1.147 (SE = 0.160), 95% CI [0.849, 0.1471].

Furthermore, an examination of the two indirect paths was performed (see Table 5). The *first* indirect effect (controlling for M2, sex, and BMI: childhood trauma → attachment anxiety → impaired reflective functioning → grazing) was statistically significant: β = 0.281 (SE = 0.077), [95% CI: 0.149; 0.451]. In contrast, the *second* indirect effect (controlling for M1, sex, and BMI: childhood trauma → attachment avoidance → impaired reflective functioning → grazing) was not statistically significant: β = 0.013 (SE = 0.014), [95% CI: −0.013; 0.044]. The total indirect effect was statistically significant: β = 0.323 (SE = 0.117), [95% CI: 0.107; 0.565]. The degree of explained variance (*R*^2^) was 36.4% (*R*^2^ = 0.364).

## Discussion

This study provides empirical support for a developmental model linking CTEs to grazing behaviors through a complex pathway involving attachment anxiety and impaired reflective functioning. The findings contribute to the growing understanding of how early relational trauma may manifest in specific patterns of disordered eating, particularly grazing, which has been increasingly recognized as a clinically significant behavior associated with emotion regulation, food addiction, addiction-like eating behaviors, and poor treatment outcomes in bariatric surgery populations (Conceição et al., 2015; Ribeiro et al., 2023; Rossi et al., 2024).

### Main findings and theoretical implications

The results confirm the hypothesized mediation model, demonstrating that the relationship between CTEs and grazing behaviors operates through a sophisticated developmental pathway rather than through direct effects – in line with other studies that have investigated different disordered eating behaviors (Musetti et al., 2023; Rossi et al., 2025a). This indirect pathway provides important insights into the psychological mechanisms underlying the emergence of repetitive, compulsive eating patterns that characterize grazing behaviors.

Central to our findings is the critical role of attachment anxiety as a mediator between childhood trauma and impaired reflective functioning (i.e., hypomentalization). Consistent with attachment theory and previous research (Bowlby, 1988; Musetti et al., 2023; Rossi and Mannarini, 2025), CTEs significantly predicted both dimensions of insecure attachment. However, only attachment anxiety – not attachment avoidance -emerged as a significant predictor of impaired reflective functioning, supporting the differential impact of attachment insecurity patterns on mentalizing capacity. This finding aligns with theoretical predictions that attachment anxiety, characterized by hypervigilance to relational threats and intense emotional responses, may be more directly disruptive to the development and maintenance of reflective functioning compared to attachment avoidance, which involves defensive emotional suppression (Sheinbaum et al., 2015; Long et al., 2020).

The absence of a direct relationship between childhood trauma and impaired reflective functioning, coupled with the significant indirect effect through attachment anxiety, underscores the importance of relational patterns in translating early adverse experiences into cognitive-emotional vulnerabilities (Midolo et al., 2020; Santoro et al., 2021). This suggests that it is not trauma per se, but rather the specific relational insecurities that trauma engenders – particularly the anxious preoccupation with relationships and fear of abandonment—that most directly compromises an individual’s capacity to understand and regulate mental states (Santoro et al., 2021, 2025b).

Also, the strong association between impaired reflective functioning and grazing behaviors represents a key finding that illuminates the psychological mechanisms underlying this specific form of disordered eating. Impaired reflective functioning emerged as the proximal predictor of grazing, suggesting that when individuals cannot adequately understand or regulate their emotional experiences through mentalization, they may resort to disordered eating behaviors (Rossi et al., 2023b, 2025b; Rossi, 2025)—such as repetitive eating behaviors – as an alternative regulatory strategy.

This finding is consistent with contemporary theories of eating disorders that emphasize the role of emotion regulation deficits in maintaining disordered eating behaviors (Whiteside et al., 2007; Aldao et al., 2010; Tan and Chow, 2014; Luyten et al., 2020; Musetti et al., 2023). When the capacity of self-regulating is compromised, individuals may experience internal states as overwhelming and incomprehensible, leading them to seek external means of regulation (Schimmenti et al., 2022; Rossi et al., 2025b; Santoro et al., 2025b). Food, particularly in the repetitive, compulsive pattern characteristic of grazing, may serve this regulatory function by providing immediate but temporary relief from distressing emotional states (Conceição et al., 2014a; Rossi et al., 2025b).

The relationship between impaired reflective functioning and grazing may be particularly relevant to understanding the addictive-like qualities of this eating behavior. Grazing involves the repeated consumption of small amounts of food throughout the day, often in response to emotional triggers rather than physiological hunger (Conceição et al., 2014a). This pattern shares characteristics with other behavioral addictions, including loss of control, continued engagement despite negative consequences, and difficulty with behavioral cessation (Conceição et al., 2014b). The finding that impaired reflective functioning predicts grazing suggests that the addictive-like qualities of this behavior may stem from fundamental difficulties in understanding and regulating psychological states.

### Implications for understanding grazing as a clinical phenomenon

The current findings contribute to the evolving understanding of grazing as a clinically significant eating behavior. Unlike formally diagnosed eating disorders such as anorexia or bulimia nervosa, grazing represents a dimensional, transdiagnostic eating pattern not currently recognized in the DSM-5, yet it shares key features with established eating disorders (e.g., loss of control, emotional triggers, psychological distress) and bridges traditional categories of eating pathology and addiction (Conceição et al., 2014a; Gearhardt and Schulte, 2021; Gearhardt et al., 2024; Rossi et al., 2024). The identification of a developmental pathway from CTEs through attachment anxiety and mentalization deficits to grazing provides empirical support for conceptualizing grazing within a broader framework of trauma-informed approaches to eating disorders (Brewerton, 2007, 2017; Wiss and LaFata, 2025).

The moderate association between attachment anxiety and grazing (both directly and indirectly through impaired reflective functioning) has important implications for understanding the interpersonal context of this eating behavior. Individuals with attachment anxiety are characterized by negative self-views, fear of abandonment, and excessive reliance on others for emotional regulation (Bartholomew and Horowitz, 1991; Mikulincer and Shaver, 2003). When these individuals also experience deficits in reflective functioning, they may be particularly vulnerable to using food as a means of self-soothing and emotional regulation (Musetti et al., 2023; Santoro et al., 2025a). The repetitive, controllable nature of grazing (Conceição et al., 2014a, 2017) may provide a sense of comfort and predictability that is otherwise lacking in their relational experiences.

This understanding has particular relevance for clinical populations, especially those undergoing bariatric surgery. Grazing has been identified as one of the strongest predictors of poor postoperative outcomes, including insufficient weight loss and weight regain (Colles et al., 2008; Conceição et al., 2015; Mechanick et al., 2020). The current findings suggest that these poor outcomes may not simply reflect “behavioral failures” but rather represent manifestations of deeper psychological vulnerabilities rooted in early relational trauma (Teicher et al., 2022) and ongoing difficulties with emotional regulation (Asen and Fonagy, 2017).

### Differential effects of attachment dimensions

The finding that attachment anxiety, but not attachment avoidance, mediates the relationship between trauma and impaired reflective functioning has important theoretical and clinical implications. This differential pattern suggests that different forms of attachment insecurity may be associated with distinct vulnerabilities and coping strategies (Mikulincer et al., 2003; Jewell et al., 2023).

Individuals with high attachment avoidance may maintain some capacity for reflective functioning through emotional distancing and cognitive suppression, even in the context of trauma history. While this defensive strategy may preserve some mentalizing abilities, it likely comes at the cost of emotional authenticity and interpersonal connection (Davila et al., 2005). In contrast, individuals with high attachment anxiety may experience emotional flooding that overwhelms their capacity for reflection, particularly during times of stress or interpersonal threat (Bateman and Fonagy, 2019; Luyten et al., 2020; Rossi and Mannarini, 2025).

This differential vulnerability has implications for understanding eating disorder subtypes and treatment approaches (Jewell et al., 2016; Tasca, 2019). The pathway from trauma through attachment anxiety to grazing may be particularly relevant for understanding eating disorders characterized by emotional eating and loss of control (Heriseanu et al., 2019; Ribeiro et al., 2023), while other pathways involving attachment avoidance may be more relevant for restrictive eating patterns or disorders characterized by emotional suppression (Solmi et al., 2020).

### Strengths and limitations

Several limitations should be acknowledged in interpreting these findings. The cross-sectional design precludes definitive causal inferences, although the theoretical model is grounded in developmental theory that suggests temporal ordering of variables (Bowlby, 1973; Fonagy et al., 2002). Longitudinal research would be valuable for confirming the proposed developmental sequence and identifying critical periods for intervention. The reliance on self-report measures introduces potential biases, particularly for sensitive topics such as childhood trauma and eating behaviors (Pozzar et al., 2020). The retrospective assessment of childhood experiences may be influenced by current psychological state and memory biases (Bernstein et al., 2003). Furthermore, the use of self-report measures for assessing attachment may be particularly problematic for individuals high in attachment avoidance, whose characteristic deactivating strategies – including emotional suppression, defensive self-reliance, and minimization of distress – may lead to underreporting of psychological difficulties and attachment-related concerns (Dozier and Lee, 1995; Edelstein and Shaver, 2004). Such response biases could contribute to the absence of significant associations between attachment avoidance and other study variables, and future studies should consider incorporating multi-method assessment approaches, including observational or interview-based measures, to complement self-report data. Moreover, regarding the assessment of reflective functioning, the present study utilized only the uncertainty subscale of the RFQ, which measures hypomentalization. This methodological choice was guided by both theoretical and empirical considerations. First, hypomentalization represents the predominant pattern of impaired reflective functioning in the context of attachment insecurity and eating pathology (Rossi et al., 2025a, 2025b; Santoro et al., 2025a), whereas hypermentalization appears less central to these dynamics. Second, recent psychometric investigations have raised concerns about the structural validity of the RFQ’s certainty subscale, with some studies suggesting that the instrument may primarily capture a unidimensional construct focused on hypomentalization (Müller et al., 2022; Horváth et al., 2023). Nevertheless, future research should consider assessing both dimensions of reflective functioning impairment to provide a more comprehensive understanding of mentalizing difficulties in relation to eating behaviors. Additionally, the predominantly female sample (81.2%) limits generalizability to male populations, although this distribution reflects the higher prevalence of eating disorders and related behaviors among women (Solmi et al., 2020) and women’s greater propensity to participate in survey research (Smith, 2008). The study’s focus on a specific set of mediating variables (attachment dimensions and reflective functioning) represents both a strength and limitation. While this focused approach allows for detailed examination of theoretically grounded pathways, other potentially important factors such as emotion regulation strategies, social support, genetic vulnerabilities, or specific trauma characteristics were not included (Panzeri et al., 2022, 2024a; Teicher et al., 2022).

However, despite these limitations, this study presents several notable strengths that enhance confidence in the findings and their clinical applicability. First, the use of structural equation modeling with latent variables provides robust statistical testing of the hypothesized mediational pathways while accounting for measurement error (Kline, 2023). The implementation of item parceling helps reduce the impact of measurement error and improves model fit (Little et al., 2013). Second, the large sample size (N = 681) provides adequate statistical power for detecting the hypothesized effects and testing complex mediational models. Third, this study is grounded in well-established theoretical frameworks from attachment theory (Bowlby, 1973; Cassidy and Shaver, 2018), mentalization theory (Fonagy et al., 2002), and eating disorder research, providing strong conceptual foundations for the tested model. Fourth, the study employed rigorous data quality screening procedures to ensure the validity of online responses (Pozzar et al., 2020), enhancing the reliability of the findings. Fifth, the use of validated Italian versions of all instruments (Sacchi et al., 2018; Morandotti et al., 2018; Rossi et al., 2024) ensures cultural appropriateness and psychometric adequacy for the study population. Finally, the focus on grazing as a specific eating behavior fills an important gap in the literature, as this behavior has been understudied despite its clinical significance in bariatric surgery populations (Colles et al., 2008; Mechanick et al., 2020).

### Clinical implications and treatment considerations

The findings have several important implications for clinical assessment and treatment of individuals presenting with grazing behaviors and related eating problems. The identification of a developmental pathway from childhood trauma through attachment anxiety and mentalization deficits suggests that effective treatment approaches should address these underlying vulnerabilities rather than focusing solely on eating behaviors (Allen et al., 2008; Bateman and Fonagy, 2019).

Comprehensive assessment should include careful evaluation of trauma history, attachment patterns, and mentalizing capacity (Tasca, 2019; Jewell et al., 2016). Clinicians should be particularly attentive to the presence of attachment anxiety and reflective functioning difficulties in individuals with grazing behaviors, as these may represent important treatment targets (Cucchi et al., 2018; Kjaersdam Telléus et al., 2024).

The central role of impaired reflective functioning suggests that mentalization-based interventions may be particularly beneficial for individuals with grazing behaviors and trauma histories (Luyten et al., 2020). Mentalization-Based Treatment (MBT) has demonstrated effectiveness for various trauma-related conditions and could be adapted specifically for eating disorder populations (Bateman and Fonagy, 2019). Such interventions would focus on enhancing the capacity to understand and regulate mental states, thereby reducing reliance on food as an external regulator (Fonagy et al., 2002; Asen and Fonagy, 2017).

The prominence of attachment anxiety in the developmental pathway also suggests that interventions addressing interpersonal difficulties and relationship patterns may be crucial (Mikulincer and Shaver, 2003). Approaches that combine mentalization-based techniques with attachment-focused interventions may be particularly effective for individuals with trauma histories and grazing behaviors (Jewell et al., 2016, 2023).

Additionally, the findings support the importance of trauma-informed care in eating disorder treatment settings (Brewerton, 2007, 2017). Recognition that grazing behaviors may represent attempts at emotional regulation in the context of trauma history can help reduce stigma and blame while promoting more compassionate and effective treatment approaches (Teicher et al., 2022).

### Future research directions

Several important directions for future research emerge from these findings. Longitudinal studies following individuals from childhood through adulthood would provide more definitive evidence regarding the temporal sequencing of the proposed pathway and could identify critical periods for intervention (Linden and LeMoult, 2022).

Research examining potential moderators and additional mediators of the identified pathway would enhance understanding of individual differences in vulnerability and resilience. Factors such as social support, therapeutic intervention, positive relationship experiences, or genetic variations in stress response systems might influence the strength of associations between trauma, attachment, mentalization, and eating behaviors (Thompson, 2024).

Investigation of sex differences in the trauma-to-grazing pathway would be particularly valuable given the gender imbalance in the current sample and known sex differences in trauma responses, attachment patterns, and eating behaviors (Emery et al., 2021; Musetti et al., 2023). Moreover, future research should investigate whether the hypothesized pathways linking childhood trauma, attachment, and reflective functioning to grazing behaviors operate similarly across different eating disorder diagnoses, which would clarify whether these mechanisms are transdiagnostic or diagnosis-specific.

Clinical research examining the effectiveness of mentalization-based and attachment-focused interventions specifically for grazing behaviors would provide important evidence regarding the clinical utility of the theoretical model. Such research could examine whether improvements in mentalization capacity lead to reductions in grazing behaviors and whether attachment security can be enhanced through therapeutic intervention (Kjaersdam Telléus et al., 2024). Additionally, given the strong associations between CTEs, attachment anxiety, and grazing behaviors observed in our findings, interventions focused on self-forgiveness may represent a valuable complementary approach (Worthington et al., 2006; Worthington and Sandage, 2016; Consoli et al., 2020). Research indicates that individuals with eating disorders exhibit significantly lower levels of self-forgiveness compared to healthy controls (Lander, 2012; Watson et al., 2012). Self-forgiveness interventions may help reduce the persistent self-blame and shame that often underlie disordered eating patterns, particularly among trauma survivors. By fostering self-compassion, such approaches could enhance treatment outcomes and facilitate healthier emotional regulation strategies.

Finally, research examining the relationship between grazing and other forms of disordered eating, as well as its specific role in food addiction frameworks, would contribute to better nosological understanding and treatment development (Ribeiro et al., 2023; LaFata et al., 2025).

## Conclusion

This study provides the first empirical examination of a comprehensive developmental model linking childhood traumatic experiences to grazing behaviors through attachment anxiety and impaired reflective functioning. The findings highlight the importance of early relational experiences in shaping psychological vulnerabilities that may manifest as specific patterns of disordered eating in adulthood. The identification of attachment anxiety and impaired reflective functioning as key mediators in this pathway has significant theoretical and clinical implications. These findings support the growing recognition that eating disorders and related behaviors such as grazing are best understood within a broader framework that considers early relational trauma, attachment patterns, and fundamental capacities for emotional regulation. From a clinical perspective, the results underscore the importance of trauma-informed, mentalization-based approaches to treating grazing behaviors and related eating problems. Rather than focusing solely on behavioral symptoms, effective interventions should address the underlying psychological vulnerabilities that contribute to these patterns. The research contributes to the evolving understanding of grazing as a clinically significant phenomenon that bridges traditional boundaries between eating disorders and addictive behaviors. By demonstrating shared developmental pathways with other trauma-related conditions, this work supports more integrated approaches to conceptualizing and treating problematic eating behaviors. Ultimately, this investigation advances both theoretical understanding and clinical practice by providing empirical support for a comprehensive developmental model of grazing behaviors. The findings underscore the importance of addressing underlying trauma and attachment vulnerabilities in treating eating-related problems and suggest that effective interventions should target mentalization capacity and emotional regulation skills rather than focusing exclusively on eating behaviors themselves.

## Data Availability

The datasets presented in this article are not readily available because the data that support the findings of this study are available on reasonable request from the corresponding author. Requests to access the datasets should be directed to a.rossi@unipd.it.

## Footnotes

1 Different SEMs were tested varying the assumed alpha coefficient ranging from 1 (i.e., assuming no measurement error) to 0.60. The results did not show substantial changes across the models.
