# Supplementary material for: Insecure attachment and impaired reflective functioning mediate the association between childhood trauma and grazing behavior
Source: Front Psychol. 2025 Dec 10;16:1703521. doi: 10.3389/fpsyg.2025.1703521 (PMC12727628; doi:10.3389/fpsyg.2025.1703521)
Supplement: Supplementary file 1 [file Data_Sheet_1.pdf]

Insecure Attachment and Impaired Reflective Functioning Mediate the Association Between Childhood Trauma and Grazing

Alessandro Alberto Rossi, Andrea Tagliagambe, Stefania Mannarini, Laura Dalla Ragione

SUPPLEMENTARY MATERIAL

Corresponding author
Alessandro Alberto Rossi, PhD
Department of Philosophy, Sociology, Education, and Applied Psychology, Section of Applied Psychology, University of Padova, Padova, Italy.
Via Venezia 12, 35131, Padua (PD), Italy
Email: a.rossi@unipd.it

SUPPLEMENTARY TABLE S1

Theoretical Pathway	Key Constructs	Representative Studies	Main Findings
**Childhood Traumatic Experiences (CTEs) → Mental Health & Eating Pathology**	CTEs (emotional abuse, neglect, physical abuse)<br><br>Eating disorders<br><br>Disordered eating	Brewerton (2007, 2017); Backholm et al. (2013); Vachon et al. (2015); Emery et al. (2021); Linden & LeMoult (2022); Teicher et al. (2022); Musetti et al. (2023); Wiss & LaFata (2025)	CTEs constitute risk factors for eating disorders and disordered eating. Emotional maltreatment shows particularly strong associations. Effects accumulate and persist into adulthood. Prevalence: ~33% of populations worldwide.
**CTEs → Insecure Attachment**	CTEs<br><br>Attachment anxiety<br><br>Attachment avoidance	Bowlby (1973, 1988); Bartholomew & Horowitz (1991); Schimmenti & Caretti (2016); Schimmenti (2018); Cassidy & Shaver (2018); Midolo et al. (2020)	Early caregiver-child interactions establish foundational relational templates. CTEs significantly increase likelihood of insecure attachment patterns. Attachment system functions as primary motivational framework for seeking protection and comfort.
**Attachment Dimensions: Differential Regulatory Strategies**	Attachment anxiety (hyperactivating strategies)<br><br>Attachment avoidance (deactivating strategies)<br><br>Reflective functioning<br><br>Emotion regulation	Mikulincer et al. (2003); Sheinbaum et al. (2015); Tasca (2019); Long et al. (2020); Luyten et al. (2020); Musetti et al. (2022, 2023); Jewell et al. (2023)	Anxiety: hyperactivating strategies → emotional flooding → directly overwhelms mentalizing.<br><br>Avoidance: deactivating strategies → emotional suppression → pseudomentalizing (superficial understanding lacking affective grounding).<br><br>Attachment anxiety more directly disruptive to RF than avoidance.
**Attachment Anxiety → Eating Pathology**	Attachment anxiety<br><br>Eating disorder severity<br><br>Self-regulation impairments	Meltzer-Brody et al. (2011); Tasca et al. (2013); Jewell et al. (2016, 2023); Tasca (2019); Solmi et al. (2020); Midolo et al. (2020); Musetti et al. (2023); Santoro et al. (2025b)	Attachment anxiety accounts for relationship between traumatic experiences and eating pathology severity. Consistently identified as vulnerability factor for self-regulation impairments and eating difficulties.

**Attachment → Reflective Functioning (RF)**	Insecure attachment (particularly anxiety)<br><br>Reflective functioning<br><br>Mentalization<br><br>Hypomentalization	Fonagy et al. (1991, 1998, 2002, 2016); Allen et al. (2008); Katznelson (2014); Bateman & Fonagy (2019); Luyten et al. (2020); Musetti et al. (2023); Rossi et al. (2025a, 2025b); Santoro et al. (2025a)	RF operationalizes mentalization: ability to understand behavior through mental states (emotions, thoughts, motivations).<br><br>Impairments: hypomentalization (excessive uncertainty) vs. hypermentalization (excessive certainty).<br><br>Hypomentalization shows robust associations with attachment insecurity and eating pathology.
**Reflective Functioning → Emotional Regulation & Adaptation**	Reflective functioning<br><br>Emotional regulation<br><br>Psychological adaptation<br><br>Maladaptive coping	Fonagy et al. (1998, 2002); Cicchetti & Toth (2005); Allen et al. (2008); Macintosh (2013); Asen & Fonagy (2017); Lorenzini et al. (2019); Garon-Bissonnette et al. (2023)	Optimal RF enables sense-making of behaviors and serves as crucial component of emotion regulation.<br><br>Impaired RF (hypomentalization) → difficulties understanding mental states → defensive withdrawal from reflection → compromised emotion regulation → vulnerability to maladaptive coping.
**Reflective Functioning → Eating Behaviors**	Impaired RF (hypomentalization)<br><br>Childhood maltreatment<br><br>Attachment patterns<br><br>Problematic eating behaviors	Cucchi et al. (2018); Gagliardini et al. (2020); Santoro et al. (2021); Musetti et al. (2023); Sarig-Shmueli et al. (2023); Gagliardini et al. (2024); Kjaersdam Telléus et al. (2024); Rossi et al. (2025a); Rossi & Mannarini (2025); Santoro et al. (2025a)	Uncertainty about mental states partially mediates relationship between childhood maltreatment, attachment, and eating behaviors.<br><br>Elevated impaired RF in eating disorders vs. controls.<br><br>When individuals cannot regulate emotions due to impaired RF, they turn to food as alternative coping strategy.

**Grazing: Characterization & Clinical Significance**	Compulsive grazing<br><br>Non-compulsive grazing<br><br>Food addiction<br><br>Loss of control<br><br>Emotion regulation	Poole et al. (2005); Colles et al. (2008); Conceição et al. (2014a, 2014b, 2015, 2017, 2023); Heriseanu et al. (2019); Gearhardt & Schulte (2021); Parnarouskis & Gearhardt (2022); Ribeiro et al. (2023); Rossi et al. (2023a, 2024); Gearhardt & DiFeliceantonio (2023); LaFata et al. (2025)	Grazing: unplanned, repetitive intake of small-moderate amounts outside structured meals.<br><br>Compulsive grazing: irresistible urge to eat, loss of control, associated with negative affect, anxiety, depression, higher BMI, poor bariatric outcomes.<br><br>Not DSM-5 disorder but clinically significant dimensional behavior.<br><br>Strongly related to food addiction and addictive-like eating.
